# Comparing internal and external validation in the discovery of qualitative treatment-subgroup effects using two small clinical trials

**DOI:** 10.1016/j.conctc.2019.100372

**Published:** 2019-04-28

**Authors:** Maren K. Olsen, Karen M. Stechuchak, Karen E. Steinhauser

**Affiliations:** a Center for Health Services Research in Primary Care, Durham Veterans Affairs Medical Center, USA; b Department of Biostatistics and Bioinformatics, Duke University School of Medicine, USA; c Department of Population Health Science, Duke University School of Medicine, USA; d Duke Palliative Care, Duke University Health System, USA

## Abstract

In a two-arm randomized trial where both arms receive active treatment (i.e., treatments A and B), often the primary goal is to determine which of the treatments, on average, is more effective. A supplementary objective is to understand possible heterogeneity in the treatment effect by identifying multivariable subgroups of patients for whom A is more effective than B and, conversely, patients for whom B is more effective than A, known as a qualitative interaction. This is the objective of the qualitative interaction trees (QUINT) algorithm developed by Dusseldorp et al (Statistics in Medicine, 2014). We apply QUINT to a small randomized trial comparing facilitated relaxation meditation to facilitated life completion and preparation among patients with life-limiting illness (n = 135). We then conduct an internal validation of the QUINT solution using bootstrap resampling and compare it to an external validation with another, similarly conducted small randomized trial. Internal and external validation showed the apparent range in effect sizes was over-estimated, and subgroups identified were not consistent between the two trials. While the qualitative interaction trees algorithm is a promising area of data-driven multivariable subgroup discovery, our analyses illustrate the importance of validating the solution, particularly for trials with smaller numbers of participants.

## 1 Introduction

The primary objective of randomized clinical trials (RCTs) is to estimate the average treatment effect. There has been increased attention to estimating heterogeneity of treatment effects, where certain patient subgroups may show greater benefit than the average treatment effect. Traditionally, subgroup analyses have been conducted via one-at-a-time subgroup by treatment interactions. While conceptually easy to understand, one-at-a-time subgroup analyses may be problematic for multiple reasons, including risk for false positives and false negatives [1]. In response, researchers proposed approaches to subgroup identification focused on general principles or guidelines, where subgroups should be pre-specified and theoretically meaningful [1,2]. Yet, conducting one-at-a-time subgroup analyses may mischaracterize the actual multivariable risk and/or benefit, which is challenging to uncover with these standard methods.

Data-driven subgroup identification methods instead approach subgroup identification from a model selection perspective [3]. Many of these data-driven methods have been derived from frameworks of recursive partitioning or random forests, which are statistical classification methods that lend themselves well to exploratory analyses with many potential patient characteristics that could define subgroups [3,4]. When applied to RCTs, the goal is to identify subgroups of patients for whom the treatment effect differs across subgroup status --- e. g., subgroups of patients that benefit most from treatment. Importantly, these methods also need to account for the problematic areas of multiple testing, potential over complexity, appropriate uncertainty estimation, and reproducibility of the subgroups [3,5].

We focus on the specific situation of a RCT with two active treatment arms (A and B), where the goal is to identify subgroups where treatment A leads to improved outcomes and, conversely, also identify subgroups where treatment B leads to improved outcomes. This is known as a *qualitative* treatment-subgroup interaction. While there are numerous methods that have been developed for discovering subgroups in RCTs (e.g., see overview in Lipkovich et al. [3]), we chose to focus on a method that seeks to identify subgroups which best addresses our question of “which treatment works for whom”. Answering this question is of greatest interest for optimal treatment assignment when two active treatments are being compared.

In their methodology called QUINT (QUalitative Interaction Trees), Dusseldorp and Mechelen developed a sequential partitioning method for identifying subgroups exhibiting qualitative interactions [6,7]. QUINT identifies whether or not qualitative subgroup effect are present, and if so, yields a tree solution which partitions the entire group of patients into three types of subgroups: treatment A is better than B; treatment B is better than A; and, neither treatment is better. QUINT searches for subgroups with the greatest absolute mean or effect size difference in treatment for a continuous outcome. A natural question is to investigate the generalizability or external validity of this solution, which is rarely possible to formally assess. Generalizability in this situation is two-fold. First, are the variables and their cutpoints defining the subgroups with treatment-effect differences stable across patient samples? Second, are the treatment difference effect sizes in these subgroups of similar magnitude across patient samples? We would expect deterioration in the performance of a fitted model compared to what would be observed by chance [8], known as optimism. For smaller trials (N ≤ 400), the extent of optimism can be estimated by validating the solution using an external sample or bootstrap resampling [6].

In this manuscript, we summarize and apply the QUINT methodology in a two-arm RCT of 135 patients. We then examine the validity of the trial's QUINT solution using bootstrap resampling. We also exploit a unique opportunity to compare internal validity metrics with external validity metrics as the two active treatment arms in the trial were also implemented in a RCT of a completely different sample of 149 patients. To our knowledge, this is the first analysis to present and compare both internal and external validation of a methodology for discovering qualitative treatment interactions in a small RCT.

## 2 Background

The Outlook intervention encourages patients with life-limiting illness to grapple with issues of preparation and life completion to improve quality of life and well-being [9,10]. In a randomized trial completed in 2013, enrolled patients from an academic medical center (n = 135) with at least one of three life-limiting illnesses (stage IV metastatic cancer, congestive heart failure, or end-stage renal disease) were randomized to receive either the Outlook intervention or a relaxation meditation (RM) control condition. Patients randomized to the Outlook intervention group met with a facilitator three times over one month for approximately 45 minutes each visit, discussing topics including life review, forgiveness, and future goals and legacy. Patients randomized to the RM group met with a facilitator three times over one month for approximately 45 minutes each visit, listening to a relaxation CD. Patients’ outcomes were assessed at baseline and five and seven weeks post baseline. The outcomes which are the focus in this manuscript include baseline to five weeks in symptoms of anxiety and quality of preparation for end of life. Throughout the manuscript, we will refer to this trial as the “Original study”.

Members of our study team also completed, in 2014, a different randomized trial with similar life-limiting illness inclusion criteria, where enrolled patients were from a Veterans Affairs medical center [11] in the same geographic location and primarily had Stage IV metastatic cancer, congestive heart failure, chronic obstructive pulmonary disease, and end-stage renal disease. In this trial, 221 patients were randomized in a 1:1:1 ratio to one of three groups: 1) the Outlook intervention; 2) the RM control; and, 3) usual care. For the purpose of our analyses in this manuscript, patients randomized to usual care were removed from the sample, leaving n = 149 patients randomized to either Outlook or RM. The Outlook and RM interventions were also delivered in three 45-min sessions over a one month period, and the outcomes and timing of assessments were identical to the original study. Throughout the manuscript, we will refer to this trial as the “External study”.

The trial investigators considered both the Outlook intervention and RM as active conditions. Results for both the Original and External studies indicated some, although generally not statistically significant, within-arm improvement in outcomes from baseline to post-intervention, and these small to moderate improvements were seen in patients randomized to both of these conditions. Symptoms of anxiety were assessed via the brief POMS measure [12]; in the Original study, patients randomized to Outlook had a mean improvement of 0.35 (95% CI: −0.63, 1.33), and patients randomized to RM had a mean improvement of −0.18 (95% CI: −1.12, 0.77). In the External study, patients randomized to Outlook had a mean improvement of 0.31 (95% CI: −0.55, 1.17), and patients randomized to RM had a mean improvement of 0.26 (95% CI: −0.61, 1.13). Quality of preparation for end-of-life was measured with the QUAL-E [13]; in the Original study, patients randomized to Outlook had a mean improvement of 0.38 (95% CI: −0.50, 1.27), and patients randomized to RM had a mean improvement of 0.79 (95% CI: −0.06, 1.65). In the External study, patients randomized to Outlook had a mean improvement of 0.67 (95% CI: 0.02, 1.31), and patients randomized to RM had a mean improvement of 0.90 (95% CI: 0.23, 1.56). There were no differences between the two active arms for either of these outcomes [11]. Consequently, with the goal of optimizing treatment assignment for this patient population, there was further interest to understand if there were certain types of patients who had improved outcomes in Outlook compared to RM, and, conversely, if there were other types of patients who had improved outcomes with RM compared to Outlook.

## 3 Methods

Detecting this specific type of treatment-subgroup interaction, namely a qualitative interaction, is the primary objective of QUINT. The QUINT algorithm, a type of recursive partitioning, is described in detail by Dusseldorop and Mechelen (2014) in their original publication. We provide a brief summary here. In QUINT, the outcome variable is assumed to be continuous; for the purposes of our illustration, we define the outcome to be the baseline to post-treatment change score. The goal is to search for subgroups as defined by baseline variables that yield the largest (or approximately equal) treatment differences on the absolute mean difference scale. With QUINT, the difference in treatment outcome can either be specified as the actual difference in means of the outcome variable or as an effect size defined as Cohen's *d* [14]. Dusseldorp and Mechelen (2014) generally recommend using the effect size criterion, rather than actual mean differences, because it accounts for precision or overlap in the outcome distribution between treatment A and B. An exception to this may be when the outcome scales have a clear pragmatic meaning that is of clinical interest.

If qualitative subgroup interactions are present, it is possible for QUINT to yield three types of subgroups on outcome improvement according to baseline variable splits: treatment A > treatment B; treatment A < treatment B; treatment A ≈ B. These subgroups are based on a binary tree, with subgroups, or leaves, *l* = 1, …, L. To find these subgroups, QUINT maximizes two conditions simultaneously: the ‘difference in treatment component’ and the ‘cardinality component’, which is the sum of the number of patients corresponding to the leaves assigned to each subgroup. In the implementation of QUINT, it is possible to weight these two components differentially (default option is 1:1), as defined in equation 6 of Dusseldorp and Mechelen (2014).

As a first step, QUINT searches all candidate baseline variables for an optimal split; baseline variables can either be dichotomous (and, therefore, just examines the two levels for this type of variable) or continuous, and searches across all possible values of the continuous scale for an optimal split. The split that maximizes the treatment and cardinality components defines the first split, generating two subgroups. Then, within each subgroup, the process is repeated. The QUINT algorithm stops when a split no longer yields a higher value of the treatment and cardinality component, or when a maximum number of subgroups (as specified by the user) is met. As a final step, to avoid over fitting, Dusseldorp and Mechelen recommend reducing the number of subgroups found using a bootstrap-based bias-correction procedure. The conclusion of a QUINT implementation yields the value of the baseline variable which defines the subgroups, the means for treatment A and treatment B in each subgroup, the treatment A vs treatment B effect size, and the sample size for treatments A and B.

### 3.1 QUINT specification

The QUINT algorithm is implemented with the package quint in R, a free software environment (R Core Team, 2014). Dusseldorp et al. [15] provide a detailed step-by-step guide and illustration of how to use quint. Users have the option to either use default values or modify various tuning parameters via the quint. control function (see Table 1 of Dusseldorp et al.). In our implementation, we used R version 3.3.2 and the default values of: “effect size” for the type of partitioning criterion; weights of the difference in treatment outcome and cardinality components; stopping criterion; minimal sample size in a subgroup; and minimum absolute effect size in each subgroup (*d*_min_ = 0.3). Because of our smaller sample size, and to improve stability, we increased the number of bootstrap samples from 25 to 200. Finally, for all analyses, we used the prune. quint function to automatically select the best tree.

**Table 1:** Variables included in QUINT specification for both Original and External Studies.

	Variable Name used in QUINT	Original Study N = 135	External Study N = 149
**Demographic characteristics**			
Age, mean (SD)	Age	62.5 (13.3)	68.5 (9.6)
Highest level of education is high school or less, n (%)	EDUC_HS_OR_LESS	60 (44.4)	67 (45.0)
Married, n (%)	Married	73 (54.1)	84 (56.4)
Non-Hispanic white race, n (%)	White_Race	69 (51.1)	78 (52.3)
High score on Palliative Performance Scale, n (%) [17]	HighPPS	110 (81.5)	110 (73.8)
Cancer diagnosis^a^, n (%)	CancerDX	94 (69.6)	68 (45.6)
Insecure financial situation, n (%)	InsecureFinance	59 (43.7)	65 (43.6)
Faith or spirituality is “Very important” in life^b^, n (%)	VeryImpFaith	100 (74.1)	98 (65.8)
**Baseline measures, mean (SD)**			
Functional impairment [18]	Functional_Impairment	5.8 (1.8)	6.5 (1.9)
Functional impairment (walk and groom self) [18]	Walk_Groom_Impair	2.3 (0.7)	2.3 (0.6)
Fundamental-functional impairment [18]	FundFunc_Impair	5.5 (1.1)	5.6 (1.3)
Instrumental-functional impairment [18]	Instr_Impairment	9.9 (3.1)	9.5 (3.0)
QUAL-E Preparation [13]	Baseline_Preparation	14.0 (4.0)	15.1 (3.4)
QUAL-E Life completion [13]	Baseline_LifeComp	27.3 (5.1)	26.2 (5.5)
QUAL-E Relationship with health system [13]	RelationshipHealthSystem	19.9 (3.7)	19.2 (3.7)
QUAL-E Quality of life item [13]	QualityOfLife	3.7 (0.8)	3.6 (0.8)
CES-D-10 [19]	CESD10	8.8 (6.1)	8.4 (5.6)
FACIT-Sp Faith [20,21]	Faith	11.6 (4.0)	11.1 (3.7)
FACIT-Sp Peace [20,21]	Peace	11.1 (3.4)	10.9 (3.2)
FACIT-Sp Meaning [20,21]	Meaning	12.6 (3.0)	12.0 (3.0)
Anxiety^12,^^c^	Anxiety	4.3 (4.8)	5.7 (4.3)
Daily Spiritual Experience Scale [22]	SpiritualExp	2.5 (1.1)	2.7 (1.0)
FACT-G Emotional well-being [23]	Emotional	18.5 (4.5)	19.2 (4.0)
FACT-G Physical well-being [23]	Physical	18.6 (6.2)	19.6 (5.3)
FACT-G Functional well-being [23]	Functional	15.8 (6.8)	14.4 (5.7)
FACT-G Social well-being^23,^^d^	Social	18.5 (4.8)	17.7 (4.7)

QUAL-E = a 31-item validated measure of quality of life at the end of life; CES-D = Centers for Epidemiological Study depression scale; FACIT-Sp = Functional Assessment of Chronic Illness Therapy - Spiritual Well-Being; FACT-G = Functional Assessment of Cancer Therapy –General.

Note, variables described by means (SD) are continuous variables, variables described by n (%) are dichotomous variables. SD = standard deviation.

a Original study: Cancer diagnosis vs. congestive heart failure or end-stage renal disease. External study: Cancer diagnosis vs. congestive heart failure, end-stage renal disease, chronic obstructive pulmonary disease, or end-stage liver disease.

b Faith or spiritual importance: “Very important” vs. “Somewhat” or “Not at All Important”.

c Profile of Mood States Anxiety subscale, one of 6 items not queried in survey and thus omitted from score calculation.

d Satisfaction with sex life question omitted from score calculation.

### 3.2 Original Outlook study

As mentioned above, the Original Outlook study included n = 135 patients randomized to receive either Outlook or RM. Table 1 describes sample characteristics and explanatory variables considered for the QUINT analysis. In all, 26 candidate variables were included for possible subgroup identification. The two outcomes for the QUINT analysis were constructed by calculating the difference between baseline and first follow-up at five weeks (F1). For interpretability across outcomes, a positive value of this difference always indicates improvement (i.e., for Anxiety, we calculated Baseline-F1, and for Preparation, F1-Baseline). A small number of patients did not provide complete data on the explanatory variables or dropped out of the study prior to the first follow up, so we created a single imputation of all variables using PROC MI in SAS v 9.2 (Cary, NC) to prevent varying sample sizes across models. The single imputation was generated from the mean and covariance parameter values found via the expectation maximization algorithm [16].

### 3.3 Apparent performance

Using the Original study, a QUINT analysis was conducted on both outcomes using the 26 baseline explanatory variables and tuning parameters as specified above. The best tree was then selected via the prune. quint function, which prunes the tree to an optimal number of subgroups, or leaves, defined as *L*_optimal_. From each of the final trees grown via QUINT, the *apparent performance* was calculated by taking the difference of effect sizes between the subgroup with the largest effect size and the subgroup with the smallest effect size, **range**_**app**_** = **$d_{\max}^{\mathrm{orig}}\;-\;d_{\min}^{\mathrm{orig}}$. The subgroups identified in the final tree solution for each outcome were also retained for comparison.

### 3.4 Internal Validation

Given the small sample, we then conducted an internal validation by creating 100 bootstrap samples (i.e., B = 100) from the Original study. We then executed the following five steps (as originally outlined in Section C.2 of the web appendix of Dusseldorp and Mechelen [6]) for each of the two study outcomes.

Step 1. We conducted a QUINT analysis on each of the bootstrap samples where the maximum number of subgroups, or leaves, was set to be *L*_optimal_. All other tuning parameters were identical to the Original study QUINT analysis (section 3.1 above).
Step 2. For each bootstrap sample, we identified the subgroup with the largest positive effect size ($d_{b,max}^{boot}$) and the subgroup with largest negative effect size ($d_{b,min}^{boot}$). We then calculated the mean of the difference of these effect sizes across the bootstrap samples, **mean(Range**_**boot**_**) = **$\frac{1}{B}\overset{B}{\underset{b=1}{\sum }}\left(d_{b,\max}^{\mathrm{boot}}\;-\;d_{b,\min}^{\mathrm{boot}}\right)\;\;$**.**
Step 3. With the solution from each QUINT analysis of the bootstrap samples – i.e., the subgroups identified – we then applied these subgroup definitions to the Original study and identified the subgroup with the largest positive effect size ($d_{b,max}^{orig}\;)\;$ and the subgroup with the largest negative effect size ($d_{b,min}^{orig}$). Note that we are simply interested in the range of the effect sizes, not whether or not the same subgroups defined the maximum and minimum effect sizes. We then calculated the mean of the difference of these effect sizes across the bootstrap samples, **mean(Range**_**Orig**_**) =** $\frac{1}{B}\overset{B}{\underset{b=1}{\sum }}\left(d_{b,\max}^{\mathrm{orig}}\;-\;d_{b,\min}^{\mathrm{orig}}\right)\;\;$**.**
Step 4. Using the results from Steps 2 and 3, we calculated the *expected over-optimism*, as defined by the difference in range of effect sizes: ${\bar{O}}_{\mathrm{range}}$ = **Mean(Range**_**boot**_**) - Mean(Range**_**Orig**_**).**
Step 5. As a final step, we *corrected the apparent performance* of the Original study for optimism, **range**_**appcorr**_ = **range**_**app**_ **-** ${\bar{O}}_{\mathrm{range}}$

These general steps may also be implemented in the quint. validate function, which generates the expected over-optimism (${\bar{O}}_{\mathrm{range}})\;$ if all of the bootstrap samples yield a QUINT solution.

### 3.5 External validation

We then conducted an external validation given the availability of our External study; recall that the explanatory variables, outcomes, and timing of outcomes were identical to that of the Original study. Therefore, all outcomes and explanatory variables were defined in an identical manner to that of the Original study. And, again, a single imputation was generated to ensure complete data on the explanatory variables and outcomes. The QUINT solution found for each of the two outcomes in the Original study was applied to the External study sample. Using an equivalent methodology as with the bootstrap methodology, the external performance was calculated by taking the difference of effect sizes between the subgroup with the largest effect size and the subgroup with the smallest effect size, **range**_**ex**_** =** $d_{\max}^{\mathrm{ex}}\;-\;d_{\min}^{\mathrm{ex}}$**.** This quantity provides a direct comparison to the apparent performance found in the Original study and may be thought of as an estimate of the population value in range of effect sizes.

Finally, we also conducted a de novo QUINT analysis to the External study sample to explore whether or not similar splitting variables and patterns of results would be seen as compared to the Original study QUINT analysis.

### 3.6 Sensitivity analysis

Because Dusseldorop and Mechelen [6] advise increasing the minimum effect size criteria with smaller sample sizes, we also explored the sensitivity of our results when increasing the minimum effect size needed for a qualitative interaction to be detected to 0.40.

## 4 Results

The results for the QUINT solution, internal and external validation, and sensitivity analysis are presented separately for each outcome and then summarized. The effect sizes, *d*, are calculated as RM – Outlook; so, a positive value of *d* indicates greater improvement for RM compared to Outlook and a negative value of *d* indicates greater improvement for Outlook compared to RM.

### 4.1 Anxiety

**Fig. 1:**
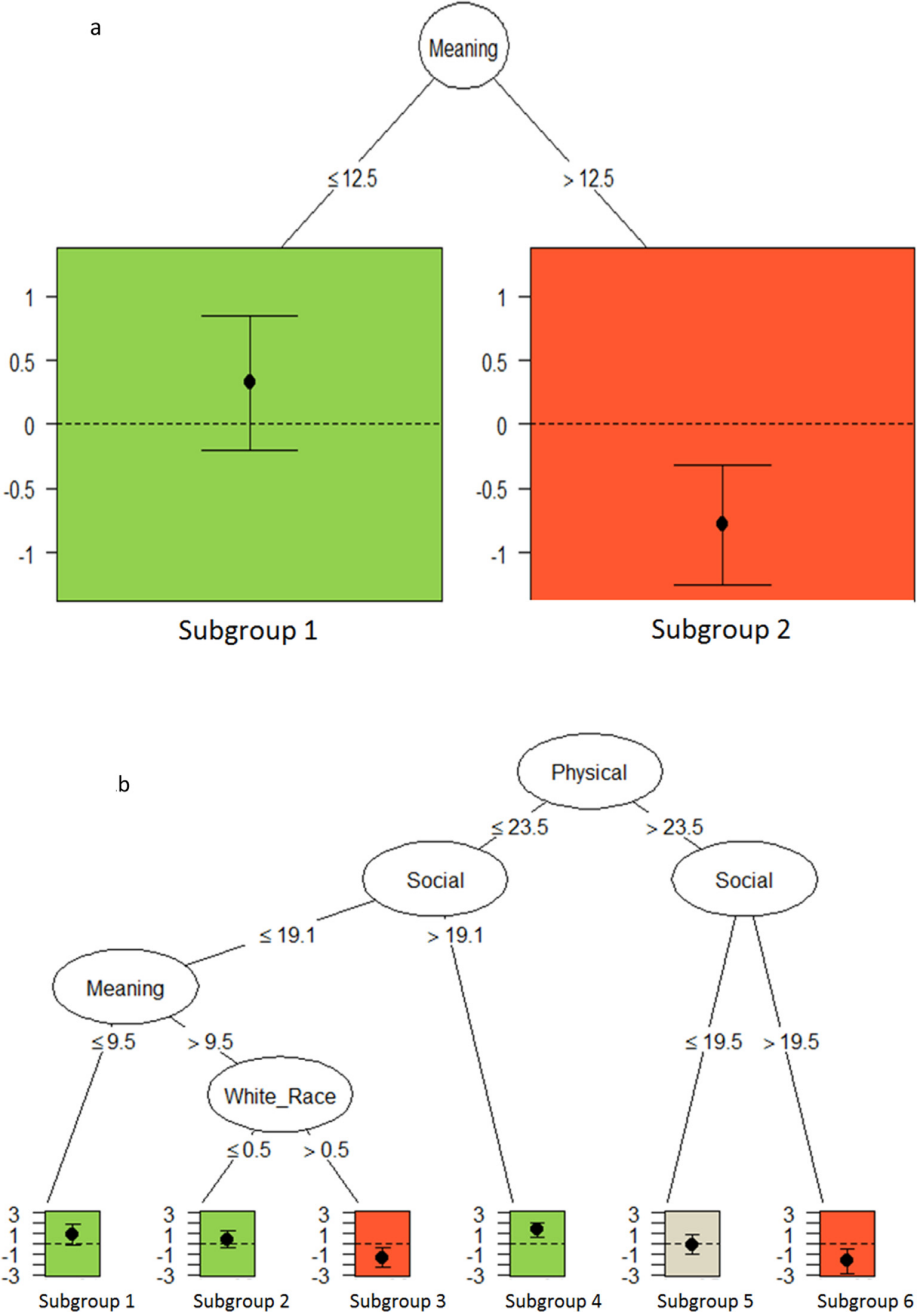
a. Anxiety: Pruned QUINT Solution in Original Study. Vertical axis represents effect size (*d*). Subgroup 1 (n = 58) had meaning scores ≤ 12.5 and showed greater improvement in anxiety in RM compared to Outlook (*d* = 0.32). Subgroup 2 (n = 77) had meaning scores > 12.5 and showed greater improvement in anxiety in Outlook compared to RM (*d* = −0.78). b. Anxiety: Pruned QUINT Solution in External Study. Vertical axis represents effect size (*d)*. The following subgroups showed greater improvement in anxiety in RM compared to Outlook: Subgroup 1 = physical well-being scores ≤23.5, social well-being scores ≤19.1, and meaning scores ≤9.5 (n = 18, *d* = 0.88); Subgroup 2 = physical well-being scores ≤23.5, social well-being scores ≤19.1, meaning scores >9.5, and were not of white race (n = 26, *d* = 0.42); Subgroup 4 = physical well-being ≤23.5, social well-being scores >19.1 (n = 41, *d* = 1.35). The following subgroups showed greater improvement in anxiety in Outlook compared to RM: Subgroup 3 = physical well-being scores ≤23.5, social well-being scores ≤19.1, meaning scores >9.5, and were of white race (n = 25, *d* = −1.37); Subgroup 6 = physical well-being scores >23.5 and social well-being scores >19.5 (n = 18, *d* = −1.71). The following subgroup for whom treatment arm did not make a difference: Subgroup 5 = physical well-being scores >23.5, social well-being scores ≤19.5 (n = 21, *d* = −0.09).

Fig. 1a displays the pruned tree final QUINT solution for improvements in Anxiety for the Original study. The pruned tree contained only two subgroups, where the spiritual well-being meaning subscale (as measured by the FACIT-Sp [20,21]) split at 12.5 met the optimal search criteria. Patients with higher meaning (>12.5) [n = 77] had greater improvements if randomized to the Outlook intervention as compared to RM (*d* = −0.78). In contrast, patients with lower meaning (≤12.5) [n = 58] had greater improvements if randomized to RM compared to Outlook (*d* = 0.32).

Internal validation results are presented in Table 2. Because the QUINT solution for Anxiety had only two subgroups, the *apparent performance* was simply the difference in the two effect sizes (*maximum – minimum*), 1.10. Of the 100 bootstrap samples, the QUINT analysis, restricted to a maximum of 2 subgroups, identified a qualitative interaction in 87 samples. The mean estimate of *over-optimism* from the 87 samples was 0.76, so the *optimism-corrected range in effect sizes* for Anxiety was 0.35, which is less than 0.6. Note that of the 87 samples with a qualitative interaction, 30 matched the Original study QUINT solution, where the two subgroups were defined by the baseline spiritual well-being meaning scale split at 12.5.

**Table 2:** QUINT solution range in effect sizes: Original study, internal validation, and external study.

Outcome	Apparent Performance^a^ (Range_app_)	Internal Mean Optimism (${\bar{O}}_{range}$)^b^	Optimism- corrected Range (Range_appcorr_)^c^	Estimated population value range in effect sizes (Range_ex_)^d^
Anxiety	1.10	0.76	0.35	0.01
Preparation	2.50	1.54	0.97	0.31

a The apparent performance is defined as the difference between the largest effect size (*d*) favoring RM and the largest effect size favoring Outlook. A larger apparent performance indicates greater separation in effects between subgroups. Because our minimum threshold effect size is 0.3, the minimum apparent performance is 0.6.

b Internal mean optimism: this quantity is derived via bootstrap samples of the original study and provides an estimate of how “over optimistic” the range in *d*'s in the original study may be.

c Optimism-corrected range: this is the difference between the apparent performance and the internal mean optimism. For our analysis, values under 0.6 suggest a lack of generalizability of the original solution (i.e., effect size favoring RM is less than 0.3 and effect size favoring Outlook is less than 0.3).

d Estimate of the population value in range of *d*'s: after creating subgroups in the external study equivalent to those identified in the original study, this is the difference between the subgroup with the largest *d* and the subgroup with the smallest *d*. A value close to the optimism-corrected range provides evidence of generalizability of the QUINT solution.

The range of effect sizes in the External Study are also shown in Table 2. Two subgroups corresponding to the QUINT solution in the Original Study were created in the External study: patients with spiritual well-being meaning >12.5 (n = 69) and patients with meaning ≤ 12.5 (n = 80). The effect sizes for these two subgroups were 0.15 and 0.14, respectively, so the range was 0.01, considerably smaller than either the apparent performance or the optimism-corrected performance.

Finally, the results of the de novo QUINT analysis of Anxiety in the External study is shown in Fig. 1b and produced markedly different subgroups. The pruned tree QUINT solution yielded six subgroups, with the initial split defined by physical well-being (as measured by the FACT-G [23]) split at 23.5. The largest effect size favoring Outlook (*d* = −1.71) was in Subgroup 6 with baseline physical well-being scores >23.5 and social well-being scores >19.5 (n = 18). Conversely, among those with baseline physical well-being ≤23.5 and social well-being scores >19.1 (n = 41, Subgroup 4), patients randomized to RM had the greatest differential improvement compared to Outlook (*d* = 1.35). It is interesting to note that treatment arm did not make a difference (*d* = −0.09) among patients with baseline physical well-being scores >23.5 and social well-being scores ≤19.5 (n = 21, Subgroup 5).

### 4.2 Preparation

**Fig. 2:**
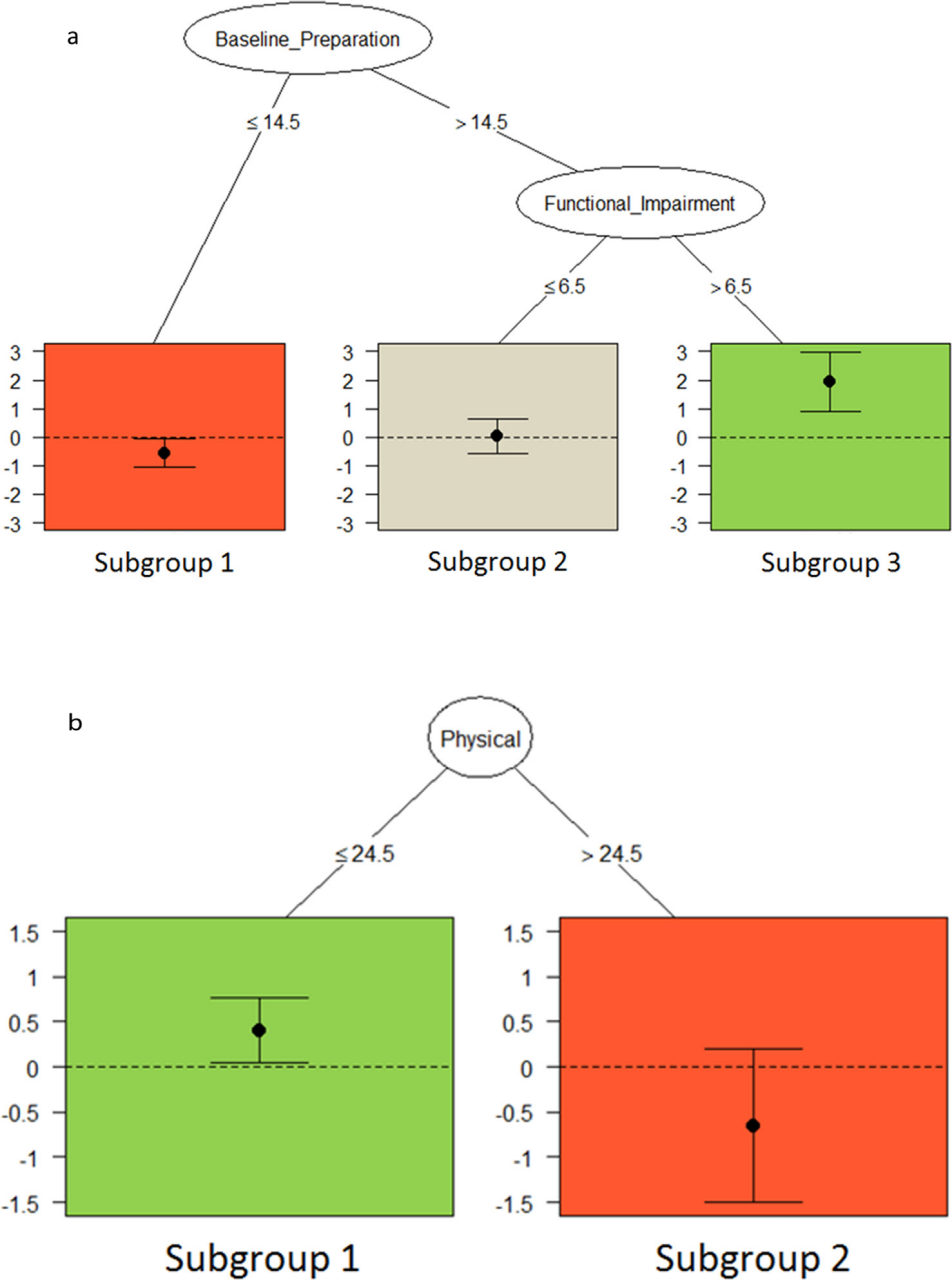
a. Preparation: Pruned QUINT Solution in Original Study. Vertical axis represents effect size (*d*). The following subgroup showed greater improvement in preparation in RM compared to Outlook: Subgroup 3 = Participants with baseline preparation >14.5 and functional impairment scores >6.5 (n = 25, *d* = 1.94). The following subgroup showed greater improvement in preparation in Outlook compared to RM: Subgroup 1 = participants with baseline preparation ≤14.5 (n = 67, *d* = −0.56). The following subgroup for whom treatment arm did not make a difference: Subgroup 2 = baseline preparation >14.5 and functional impairment scores ≤6.5, n = 43, *d* = 0.02). b. Preparation: Pruned QUINT solution in External Study. Vertical axis represents effect size (*d*). The following subgroup showed greater improvement in preparation in RM compared to Outlook: Subgroup 1 = physical well-being scores ≤24.5 (n = 124, *d* = 0.41). The following subgroup showed greater improvement in preparation in Outlook compared to RM: Subgroup 2 = physical well-being scores >24.5 (n = 25, *d* = −0.65).

Fig. 2a displays the pruned tree final QUINT solution for improvements in Preparation for the Original study; the solution has three subgroups formed by two different splitting variables. Baseline preparation, with a cutpoint of 14.5, met the optimal search criteria for the first split. Then, within the subgroup of more baseline preparation, the QUINT solution determined an optimal split on functional impairment with a cutpoint of 6.5. Patients with lower baseline preparation (≤14.5) [n = 67] had greater improvements in Preparation if randomized to the Outlook intervention as compared to RM (Subgroup 1; *d* = −0.56). In contrast, patients with more baseline preparation (>14.5) and more functional impairment (>6.5) [n = 25] had greater improvements if randomized to RM compared to Outlook (Subgroup 3; *d* = 1.94). Finally, patients with higher baseline preparation (>14.5) but less functional impairment (≤6.5) [n = 43] did not show differential improvements by intervention arm (Subgroup 2; *d* = 0.02).

Internal validation results are presented in Table 2. The *apparent performance* of 2.5 is the difference between the largest effect size (1.94) and the smallest effect size (−0.56). For the 100 bootstrap samples used in the internal validation process, when restricted to the *L*_max_ = 3 (equal to *L*_optimal_), 21 samples had no qualitative interactions found, 33 had 2 subgroups, and 46 had 3 subgroups. For those with a qualitative interaction, the mean estimate of *over-optimism* was 1.54, so the *optimism-corrected range in effect sizes* for Preparation was 0.97, which is greater than 0.6. Four of the bootstrap samples matched for both variables and cutpoints with the Original study QUINT solution. An additional 29 bootstrap samples had baseline preparation with varying cutpoints as the initial (or only) split. Sixteen bootstrap samples had instrumental-functional impairment as either the initial (or only) split, with 10 of these 16 solutions having a sole split occurring at a cutpoint of 13.5.

External validation results are presented in Table 2 and Fig. 2b. We applied the QUINT solution subgroups to the External Study: patients with baseline preparation ≤14.5 (n = 68); patients with baseline preparation >14.5 and high-functional impairment < 6.5 (n = 47); and, patients with baseline preparation > 14.5 and high-functional impairment > 6.5 (n = 34). The effect sizes were 0.14, −0.02, and 0.29, respectively. Therefore the estimate of the population value range in effect sizes was 0.31 (Table 2). Note that in both the Original and External studies, the subgroup of patients with high baseline preparation and more functional impairment led to the largest effect size favoring the RM intervention, although the effect size of 0.29 in the External Study is considerably smaller than the Original Study effect size of 1.94.

Finally, the results of the de novo QUINT analysis in the External study is shown in Fig. 2b, discovering notably different subgroups than in the Original Study (Fig. 2a). The pruned tree QUINT solution for the Preparation outcome in the External study contained only two leaves defined by FACT-G physical well-being split at 24.5. Patients with better baseline physical functioning (n = 25) who received Outlook had greater improvements in Preparation compared to RM (*d* = −0.65), whereas patients with worse physical functioning (n = 124) who received RM had greater improvements in Preparation compared to Outlook (*d* = 0.41).

### 4.3 Sensitivity analysis

A sensitivity analysis was conducted to assess results at higher minimum effect size thresholds. For improvements in Anxiety, no qualitative interactions were found for both the Original and External studies when the minimum effect size was increased to 0.40.

For improvements in Preparation, the same trees were grown for both the Original study and the External study when the minimum effect size was increased to 0.40. The apparent performance for the Original study and range of effect sizes for the External study remained the same, and the optimism-corrected range of effect sizes in the Original study decreased slightly to 0.91 while being calculated on 63 bootstrap samples with qualitative interactions.

## 5 Discussion

We present an internal and external validation analysis of two different outcomes for two small clinical trials examining the Outlook intervention compared to RM. In the QUINT analysis of anxiety symptoms in the Original Study, two subgroups were identified: patients with low baseline meaning had improved anxiety if they received RM compared to Outlook, and the converse was found for patients with high baseline meaning. The range in effect sizes was 1.10, but the optimism-corrected range was only 0.35, well below a threshold of 0.6 --- suggesting that beyond this sample, the subgroup effect sizes would be smaller than 0.3. This was affirmed when estimating the effect sizes for these subgroups in the External Study; the effect size for patients with low meaning was 0.14 and for patients with high meaning was 0.15. Additionally, the de novo QUINT solution in the External Study showed a very different pattern of treatment-subgroup effects, again showing a lack of generalizability of the QUINT solution found in the Original study.

Results from our analysis of the outcome measuring improvements in Preparation were somewhat less conclusive. In the Original Study, QUINT found three subgroups, where the largest effect size was among those patients with high baseline preparation and more functional impairment --- the RM group led to much greater improvements in Preparation between baseline and follow-up relative to Outlook. The optimism-corrected range in effect size continued to be relatively large (0.97) and was robust to the sensitivity analysis which increased in the effect size criteria. When the same subgroups were applied to the External Study, however, the effect sizes were substantially diminished, suggesting a lack of generalizability. Furthermore, our de novo analysis of the External Study found different subgroups where patients with worse physical well-being had greater improvements in preparation when receiving RM compared to Outlook. Yet, there is some similarity in the domains of more functional impairment and worse physical well-being, so future work may further investigate whether RM improves preparation for patients with more functional impairment or worse quality of life related to their physical well-being.

It is important to recognize limitations in the presented analyses. Both the Original Study and the External Studies failed to find overall differences between RM and Outlook with regard to improvements in anxiety and preparation. Also note that these subgroup analyses were purely data-driven and were not based upon a priori hypotheses. While the two studies we presented were very similar with regard to patient inclusion criteria, geographic location, and the interventions were identical, the External study enrolled predominately male patients who were Veterans. Consequently, we were not able to include patient sex as a possible candidate splitting variable. Additionally, the External study also included a usual care arm and those patients were excluded from the analyses. For consistency, we created a single imputation to handle the missing data in both of the studies. Single imputations have known limitations; areas of future work may explore congenial ways of addressing missing data within subgroup discovery methods. Finally, we only present two of several outcomes (one primary and one secondary) measured in the Original and External studies. Understanding how to discover and interpret differential treatment effects within subgroups across multiple trial outcomes is another important area of future work.

When applying QUINT to clinical trials with small sample sizes, we highly recommend conducting validation and confirming robustness of results across varying effect sizes. Caution is particularly warranted for QUINT solutions that yield smaller sample sizes in each subgroup, effect sizes, or apparent range in effect sizes. Dusseldorp and Mechelen's simulation studies also show poorer performance for sample sizes ≤ 300^6^. Our results provide concurrence with Dusseldorp and Mechelen's recommendation to increase *d*_min_ for trials with smaller sample sizes, consider setting a maximum number of subgroups to be identified, and increasing the lower bound of the minimum subgroup sample size.

The QUINT solution does not provide any measure of uncertainty (i.e., standard errors, confidence intervals) for the final solution. Therefore, we also highly recommend conducting internal validation via bootstrapping and external validation, if possible. For both outcomes, internal and external validation showed that the apparent range in effect sizes in the Original study was over-estimated. The QUINT solutions found across the bootstrap samples and the QUINT solution found with an external study de novo analysis provide additional information about the stability of the actual subgroups identified in an original QUINT analysis. The analysis presented in this paper shows that validation is a critical step when applying the QUINT algorithm to randomized trials with small sample sizes. Future work includes extending and applying these validation ideas to other data-driven subgroup identification methods, such as model-based recursive partitioning [5] or regression trees [24].
